# CD8α is expressed by human monocytes and enhances FcγR-dependent responses

**DOI:** 10.1186/1471-2172-8-12

**Published:** 2007-08-01

**Authors:** Derrick J Gibbings, Marcelo Marcet-Palacios, Yokananth Sekar, Marcus CY Ng, A Dean Befus

**Affiliations:** 1 Pulmonary Research Group, Division of Pulmonary Medicine, Department of Medicine, University of Alberta, Canada

## Abstract

**Background:**

CD8α enhances the responses of antigen-specific CTL activated through TCR through binding MHC class I, favoring lipid raft partitioning of TCR, and inducing intracellular signaling. CD8α is also found on dendritic cells and rat macrophages, but whether CD8α enhances responses of a partner receptor, like TCR, to activate these cells is not known. TCR and FcR, use analogous or occasionally interchangeable signaling mechanisms suggesting the possibility that CD8α co-activates FcR responses. Interestingly, CD8α+ monocytes are often associated with rat models of disease involving immune-complex deposition and FcR-mediated pathology, such as arthritis, glomerulonephritis, ischaemia, and tumors. While rat macrophages have been shown to express CD8α evidence for CD8α expression by mouse or human monocytes or macrophages was incomplete.

**Results:**

We detected CD8α, but not CD8β on human monocytes and the monocytic cell line THP-1 by flow cytometry. Reactivity of anti-CD8α mAb with monocytes is at least partly independent of FcR as anti-CD8α mAb detect CD8α by western blot and inhibit binding of MHC class I tetramers. CD8α mRNA is also found in monocytes and THP-1 suggesting CD8α is synthesized by monocytes and not acquired from other CD8α+ cell types. Interestingly, CD8α from monocytes and blood T cells presented distinguishable patterns by 2-D electrophoresis. Anti-CD8α mAb alone did not activate monocyte TNF release. In comparison, TNF release by human monocytes stimulated in a FcR-dependent manner with immune-complexes was enhanced by inclusion of anti-CD8α mAb in immune-complexes.

**Conclusion:**

Human monocytes express CD8α. Co-engagement of CD8α and FcR enhances monocyte TNF release, suggesting FcR may be a novel partner receptor for CD8α on innate immune cells.

## Background

CD8α is a surface glycoprotein typically found on a subpopulation of CTL [1]. CD8α enhances responses instigated through the TCR by binding MHC class I and signaling through the src kinase lck and the adaptor protein Linker for Activation of T cells (LAT) [2]. The classical co-receptor model of CD8 suggests CD8 enhances CTL activation by binding the same MHC class I-peptide as TCR [3]. Other evidence suggests CD8 is recruited to the site of T cell activation [4,5]. and can enhance T cell responses even when it does not bind at detectable levels to the same MHC class I-peptide as TCR (e.g. CD8 enhances activation of T cells with an MHC class II specific TCR [6,7]).

CD8 on T cells co-activates responses initiated by TCR, but no such co-activating role has been described for CD8 on other CD8+ cells like dendritic cells [8], NK cells [9,10]., mast cells [11] or macrophages (Mφ) [12]. Interestingly, the Fcγ chain, a component of several FcR [13], NK receptors [14], and ILT1 [15] can substitute for CD3ζ in TCR expression [16,17].; signaling [18] and T cell activation [19,20] Reciprocally, CD3ζ can substitute for Fcγ in FcR signaling [21]. Fcγ chain is an ancestral homologue of the CD3ζ chain [22]. Furthermore, CD3ζ-/-η-/- mice use Fcγ in TCR signaling and CD8-dependent CTL cytotoxicity [19], strongly suggesting CD8 can function with Fcγ in the absence of CD3ζ or η. In fact, human but not mouse mature T cells often express Syk and Fcγ alongside ZAP-70 and CD3ζ and in at least some mature effector T cells Syk and Fcγ replace ZAP-70 and CD3ζ in TCR signaling [23,24]

The cell types that express CD8α differ among mice, rats and humans. While human [9] and rat NK cells express CD8α, mouse NK cells do not [25]. Rat Mφ express CD8α [12], however, our efforts and those of others to detect CD8α protein on mouse monocytes and Mφ have been unsuccessful [26,27]. A portion of CD8α and all the CD8β found on mouse dendritic cells is derived from T cells [28]. As transfer of transmembrane proteins between cells is frequently detected, like CD8 in the case above, it is necessary to determine the source and functionality of CD8α when it is detected on a new cell type or in a new species. Since this study was started, two studies identified binding of anti-CD8α mAb at high levels to a small percentage of human monocytes during immune responses [29,30] Unfortunately neither study queried whether lower levels of CD8α were constitutively found on monocytes, demonstrated the cellular origin of the CD8α found on monocytes, or demonstrated a function for CD8α on monocytes.

In this report, we provide evidence that human monocytes express CD8α and that CD8α can enhance responses mediated through FcR.

## Results

### CD8α and not CD8β is present on human peripheral blood monocytes

**Figure 1:**
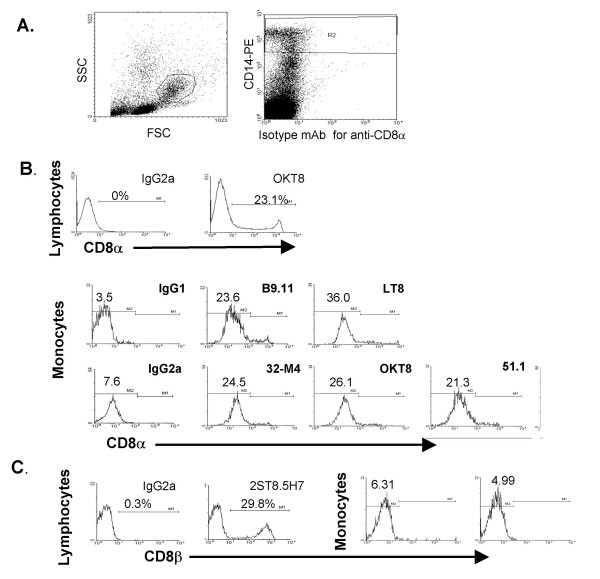
CD8α is detected by flow cytometry on CD14^hi ^monocytes from human peripheral blood. Anti-CD8α mAb bind monocytes by flow cytometry. *A*, Monocytes were gated by characteristic FSC/SSC scatter (left panel) and expression of high levels of CD14 (right panel) for all flow cytometry studies in this article. *B*, A population of lymphocytes, gated by characteristic FSC/SSC scatter, bind high levels of anti-CD8α. Blood monocytes gated in *A*, bind anti-CD8α mAb. Geometric means of monoclonal antibody binding are shown. Results are representative of five experiments. *C*, Anti-CD8β mAb bind lymphocytes but not monocytes.

Performing flow cytometry on PBMC, a subpopulation of lymphocytes (FSC/SSC gated) expressed high levels of CD8α and CD8β, as expected (Figure 1B, anti-CD8α mAb OKT8 and Figure 1C, anti-CD8β-dependent mAb 2ST8.5H7). Six anti-CD8α mAb also bound monocytes at levels greater than three times the geometric mean of isotype mAb (Figure 1B, gated for analysis by expression of high levels of CD14 [31] and characteristic FSC/SSC scatter [Figure 1A]). The monocytic cell line THP-1 bound CD8α mAb at levels comparable to blood monocytes (data not shown). CD8β was not detected on monocytes with mAb 2ST8.5H7 (Figure 1C) or 5F2 (not shown), suggesting they do not express CD8αβ (mAb 2ST8.5H7) or putative CD8ββ dimers (mAb 5F2) [32]. Accordingly, mRNA for CD8β was detected in total PBMC containing CD8αβ+ T cells, but not in highly enriched monocytes (data not shown).

### CD64 does not contribute to anti-CD8α mAb binding to monocytes

**Figure 2:**
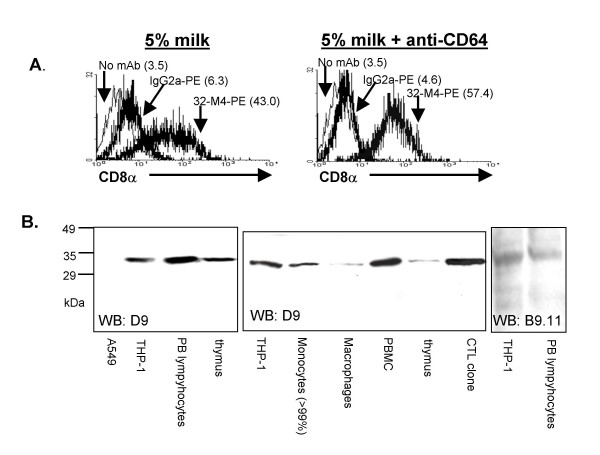
Binding of anti-CD8α mAb to monocytes is independent of FcR. *A*, Blockade of Ig binding to CD64 with anti-CD64 mAb decreases binding of isotype mAb but not anti-CD8α mAb to monocytes. Bracketed numbers are geometric means of indicated peaks. *B*, Western blot with anti-CD8α D9 detects a 32 kDa protein as expected for CD8α in THP-1, peripheral blood lymphocytes, thymus lysate, peripheral blood monocytes (>99%), GM-CSF differentiated Mφ, PBMC, thymus, and a CTL clone but not in the lung epithelial line A549 (CD8α negative control). Right, anti-CD8α mAb B9.11 detects a 32 kDa protein as expected for CD8α in THP-1 and peripheral blood lymphocytes. 1–1.5 × 10^6 ^cell equivalents were loaded in each lane.

To examine whether Fc receptors contributed to binding of anti-CD8α mAb to monocytes we first tested the contribution of CD64, the high affinity FcR. CD64 binds immunoglobulin with 100-fold or more the affinity of other FcγR [33], is the only FcγR that binds monomeric Ig [34] and preferentially binds mouse IgG2a antibodies compared to mouse IgG1 [35]. Accordingly, as human monocytes express significant amounts of CD64 [31] mouse IgG2a has a 100–1000 fold higher affinity for binding human monocytes than mouse IgG1 [36]. Incubating monocytes with human Ig preparations [37] (not shown) or a mAb which blocks binding of Ig to CD64 (clone 10.1) [38] slightly decreased binding of IgG2a isotype control mAb (11–27% geometric mean) and did not decrease binding of anti-CD8α mAb (Figure 2A).

### CD8α is detected in monocytic cell line and >99% human monocytes independent of FcR

**Figure 4:**
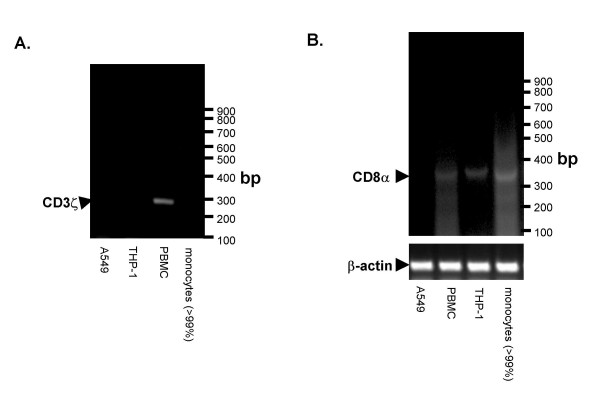
Human monocytes express CD8α mRNA. Right, purification of monocytes (>99% CD14^hi ^after sorting) was also confirmed by attempting to detect the T and NK cell transcript, CD3ζ, in monocytes. A 293 bp fragment of CD3ζ mRNA was detected in PBMC, containing T cells, but not monocytes, the monocytic line THP-1, or lung epithelial cells (A549) after 50 cycles. *B*, A 379 bp CD8α mRNA fragment was detected by RT-PCR in THP-1, >99% CD14^hi ^monocytes, PBMC, but not in a lung epithelial cell line, (A549) using intron-spanning primers, and 35 cycles of cDNA amplification. Detection of β-actin mRNA confirmed RNA extraction and RT-PCR was performed successfully.

To test whether CD8α is detected in human monocytes by a method generally acknowledged to be independent of FcγR binding to IgG we performed western blot for CD8α. Proteins at 32 kDa, consistent with CD8α were detected with anti-CD8α mAb D9 by western blot (Figure 2B) of thymus lysate, blood lymphocytes, immature monocytes (THP-1), mature *ex vivo *monocytes (>99% CD14^hi^, CD3ζ[-ve], enrichment Figure 4A), and Mφ differentiated with GM-CSF from blood monocytes, but not in lung epithelial cells (A549, negative control). Similarly, a 32 kDa protein was found by western blot with anti-CD8α mAb B9.11 in PBMC and THP-1 (Figure 2B). These data suggest that anti-CD8α mAb binding to monocytes is due to the presence of CD8α protein and not non-specific binding to FcγR.

### Peripheral localization of CD8α on human monocytes: confocal microscopy

**Figure 3:**
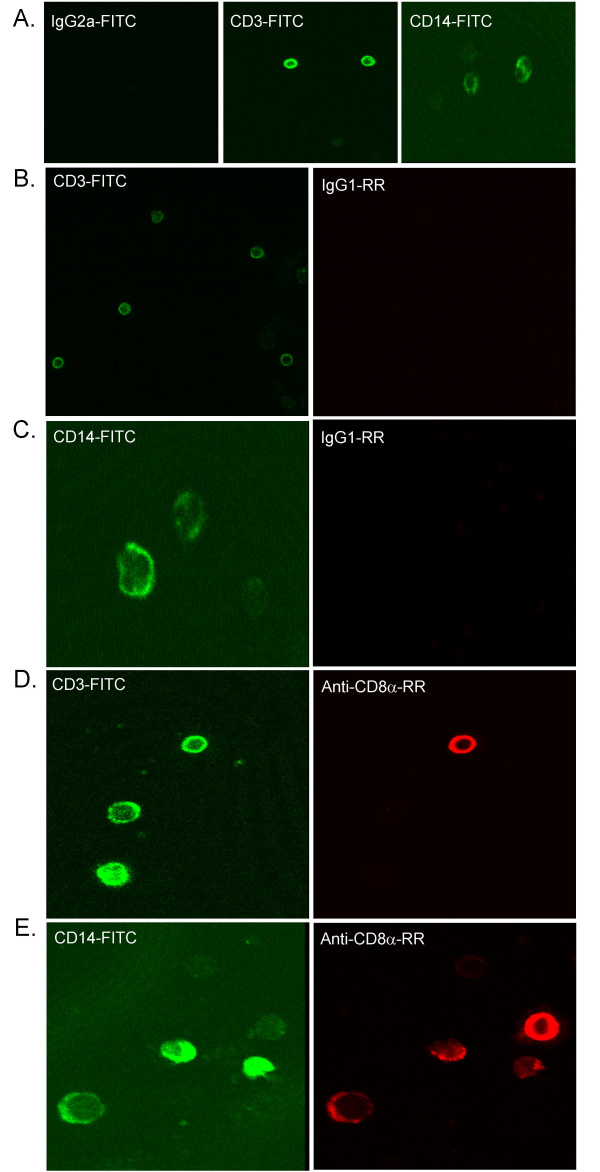
CD8α is detected by confocal microscopy on peripheral blood monocytes and lymphocytes with several anti-CD8α mAb. *A*, CD3-FITC and CD14-FITC binding to PBMC (Green). *B-E*, Anti-CD8α mAb (*D*, *E*) binding to monocytes and lymphocytes in comparison to isotype mAb (*B*, *C*) (Red). Results are representative of other anti-CD8α mAb (OKT8, 51.1, 32-M4, Nu-Ts/c, and B9.11).

To confirm the expected localization of CD8α to the cell periphery on monocytes we performed two-color confocal microscopy of permeabilized PBMC (Figure 3). All anti-CD8α mAb detected CD8α at the periphery of CD14^hi ^monocytes and some CD3^hi ^T cells (Figure 3B–E mAb B9.11 is shown, and is representative of results obtained with LT8, OKT8, 32-M4, 51.1, and Nu-Ts/c). CD8α was also observed intracellularly in some monocytes with a distribution resembling CD14, suggesting that similar to NK cells [39] a small proportion of CD8α may be found intracellularly in monocytes, perhaps in recycling endosomes.

### Human monocytes express CD8α mRNA

The monocytic cell line THP-1 must synthesize CD8α as no other sources of CD8α are available (FBS was CD8α negative by parallel western blot). In contrast *ex vivo *monocytes may acquire CD8α from other CD8α+ cells in the body. The presence of CD8α mRNA would suggest that monocytes can synthesize the CD8α protein associated with them. Due to the sensitivity of RT-PCR for mRNA from contaminating cells, we studied the cultured monocytic line THP-1 in addition to highly enriched monocytes (negative for T cell/NK cell specific CD3ζ mRNA, >99% FSC/SSC and CD14^hi ^monocytes, Figure 4A). CD8α mRNA was detected in peripheral blood mononuclear cells (containing CD8α+ T cells [positive control]), THP-1 monocytic cell line, and highly purified blood monocytes, but not in a lung epithelial cell line (A549) as expected (Figure 4B).

### CD8α on monocytes binds MHC class I

Whatever the eventual cellular derivation of CD8α protein found on *ex vivo *monocytes, its ability to function (e.g. bind MHC class I) and impact monocyte responses is practically relevant. We tested whether CD8α on human monocytes contributes to monocyte binding to MHC class I. We expected that anti-CD8α mAb would not block all tetramer binding to human monocytes because members of the immunoglobulin-like-transcript (ILT/CD85) family (ILT2, 4) expressed by monocytes bind MHC class I tetramers, interact with regions on MHC class I that overlap with CD8α and thus compete with CD8α for binding of MHC class I [40].

Thymocytes from CD8β knockout mice bind MHC class I tetramers and overexpression of CD8α enhances this CD8-dependent binding [41], suggesting that despite the heightened ability of CD8αβ (at least in an unsialylated form on thymocytes [42,43]) to bind MHC class I tetramers, CD8αα is also capable of mediating tetramer binding to T cells.

**Figure 5:**
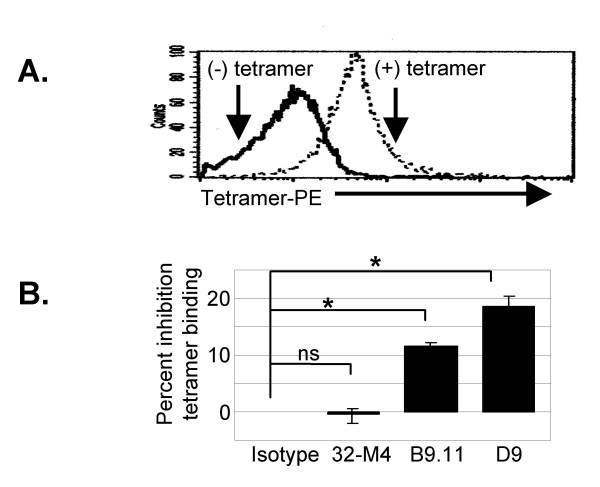
CD8α on human peripheral blood monocytes mediates MHC class I binding. *A*, Dark histogram is background fluorescence of CD14^hi ^monocytes, light histogram represents binding of PE-labeled tetramers. *B*, Bar graph is pooled results of gated CD14^hi ^monocytes from three different donors in separate experiments. Inhibition of tetramer binding is expressed as percent decrease in mean fluorescence intensity of tetramer binding due to pretreatment with anti-CD8α mAb compared to isotype mAb (* p < 0.05, non-paired t-test).

HLA-*0201 tetramers bound to nearly all CD14^hi ^monocytes (Fig 5A). Tetramers complexed with two different peptides bound similarly to monocytes (data not shown). In agreement with others [44,45]., we found that anti-CD8α clone B9.11 inhibited HLA tetramer binding (Figure 5B, 11.6%). Another anti-CD8α clone, D9, also inhibited tetramer binding (Figure 5B, 18.6%, p < 0.05). Finally, tetramer binding was not affected by clone 32-M4 (despite its ability to bind CD8α on monocytes), or isotype control mAb (Figure 5B). Other studies of MHC class I tetramer binding to CD8α using several anti-CD8α mAb have also shown that tetramer binding may be unaffected, inhibited or enhanced by anti-CD8α mAb, depending on which anti-CD8α clone is used, and TCR affinity [43,46,47].

While our evidence suggests CD8α contributes to MHC class I tetramer binding by human monocytes, the observed dimunition of tetramer binding may be via an effect on the kinetics or stability of tetramer binding to other receptors for MHC class I.

### Fewer Sialylated 34 kDa Versions of CD8α are found on Monocytes Compared to T cells

Previous publications have demonstrated notable differences in immunoprecipitated CD8α by 2-D electrophoresis. Some authors detected immunoprecipitated CD8α from thymus as restricted spots of pI ~ 6 [48]. In contrast, others detected immunoprecipitated CD8α from blood at numerous spots ranging from pI 6–9.5 [49], and molecular weights of 32 to 34 kDa [50]. We tested whether CD8α from monocytes in comparison to blood lymphocytes exhibit a distinct pattern of sialylation or other post-translational pattern detectable by 2-D electrophoresis.

**Figure 6:**
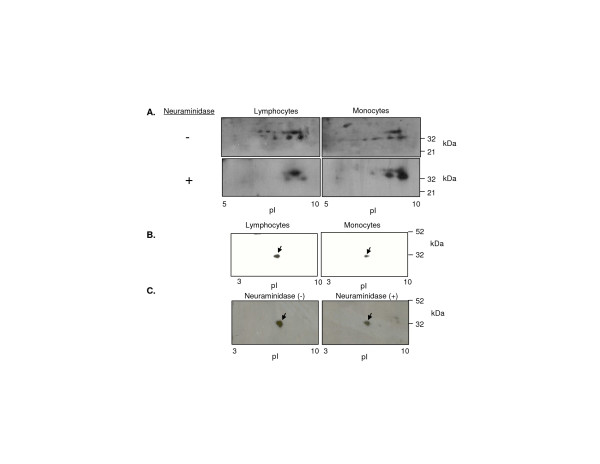
Subtle differences in CD8α between monocytes and lymphocytes detected by 2-D electrophoresis. *A*, Western blot with a polyclonal anti-CD8α antibody after 2-D electrophoresis of *ex vivo *monocytes and lymphocytes. Cell lysates of lymphocytes and monocytes from one donor were separated by adherence, halved and treated (bottom panels) or not treated (top panels) with neuraminidase before analysis. *B*, Western blot with anti-CD8α mAb D9. *C*, Neuraminidase treatment and western blot with D9 of lymphocyte lysate. Results are representative of experiments with three donors.

A polyclonal anti-CD8α antibody detected discrete spots across 2-D gels from the predicted pI of unglycosylated CD8α (~9) to pI 6–7, at Mr from 32–34 kDa (Figure 5), as shown by others [49]. Much of the heterogeneity detected with anti-CD8α polyclonal antibody could be eliminated by treatment with neuraminidase, suggesting it is due to sialylation of CD8α (Figure 6A). Notably, monocytes had less of the sialylated 34 kDa forms of CD8α than T cells in samples from three individuals (Figure 6A). In contrast, anti-CD8α clone D9 detected a single protein spot (32 kDa, ~pI 6), in a pattern similar to that found by others [48]. To confirm that the protein recognized at 32 kDa pI 6 was CD8α we sequenced it by MALDI-QTOF from 2-D gels (see Additional file 1). Neuraminidase treatment diminished but did not eliminate the protein spot recognized by anti-CD8α clone D9 and gave rise to faint basic spots similar to those observed with polyclonal anti-CD8α antibody after neuraminidase treatment. D9 may preferentially recognize particular glycosylation/sialylation patterns of CD8α (Figure 6B,C).

In our experiments large differences in 2-D electrophoresis patterns for CD8α (e.g. between D9 and polyclonal anti-CD8α Ab) are due to the specificity of different antibodies recognizing CD8α, and not a result of differences in the cell type expressing CD8α. Nonetheless, using the same polyclonal antibody, subtle differences in CD8α were found between monocytes and lymphocytes by 2-D electrophoresis.

### Anti-CD8α mAb amplifies monocyte responses to immune-complexes through FcγR

TCR and FcR use analogous and sometimes interchangeable signaling mechanisms to activate cells [20], and CD3ζη-null mice can use Fcγ to reconstitute CD8/TCR-dependent CTL cytotoxicity[23]. We investigated if CD8α on monocytes might be involved in responses to immune-complexes mediated by FcγR. To this end, we used a common immune-complex-FcγR activation system.

**Figure 7:**
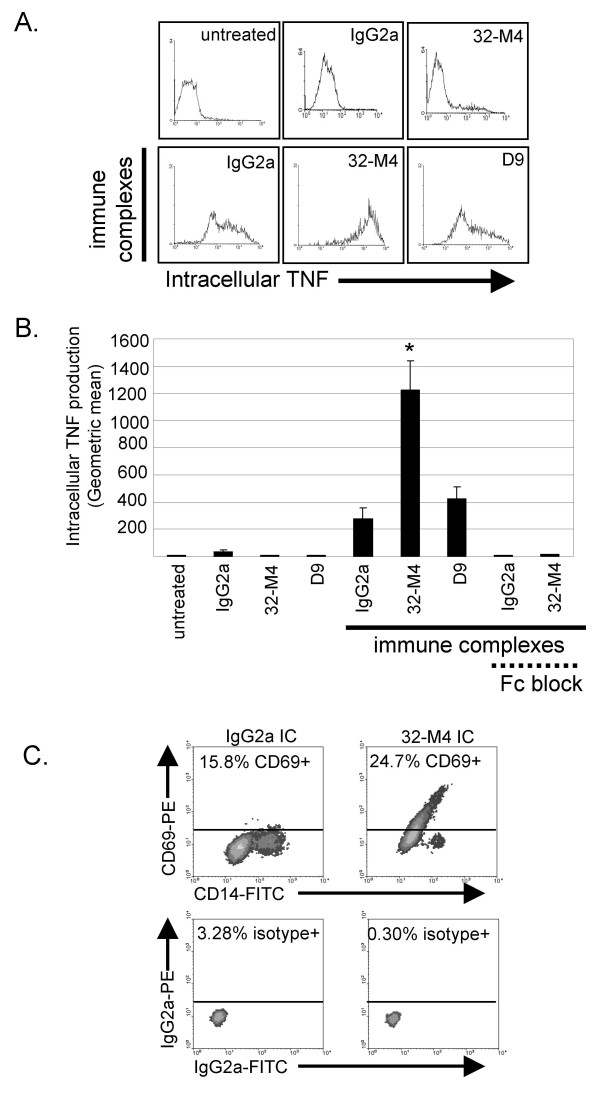
CD8α enhances monocyte TNF production in a FcγR-dependent manner. Monocytes were incubated with anti-CD8α or isotype mAb alone or in immune-complexes. Excess Fc fragment was used to block FcγR. TNF production was measured by intracellular flow cytometry after 5 h (gated on CD14^hi ^monocytes). *A*. Histograms are representative results. *B*. Bar graph represents average geometric mean of intracellular TNF from four separate experiments with monocytes from different donors. Standard error of the mean is designated by error bars. *: p < 0.05 non-paired t-test. C. CD69 and CD14 regulation induced by immune-complexes (IC) is enhanced by CD8α ligation (representative example).

Treatment of monocytes with monomeric non-specific IgG_2a _mAb induced a slight increase in monocyte TNF production (Figure 7A,B). This is likely due to the ability of the high affinity FcγR, CD64, found on the majority of monocytes to bind monomeric mouse IgG_2a _[36]. Monomeric anti-CD8α mAb did not increase monocyte TNF production more than control IgG_2a _mAb (Figure 7A,B). When monocytes were stimulated with preformed immune-complexes (isotype mAb cross-linked with anti-mouse Ig), CD14^hi ^monocytes produced moderate amounts of intracellular TNF (Figure 7A,B). Formation of immune-complexes with anti-CD8α mAb (32-M4 cross-linked with anti-mouse Ig) rather than isotype mAb, resulted in production of 2-fold more TNF by monocytes (Figure 7A,B). Immune-complexes containing anti-CD8α mAb D9 did not significantly increase monocyte TNF production above control immune-complexes, indicating that monocyte TNF release is not significantly stimulated by any immune-complex containing a mAb that binds to the monocyte surface. It is not surprising that only one of two anti-CD8α mAb enhanced FcγR-dependent responses, because when others have screened several anti-CD8α mAb in parallel for effects on CTL cytotoxicity, or CD8-MHC class I binding (see above), the effect ranged from substantial inhibition to no effect depending on the particular anti-CD8α mAb clone [51-53]

Enhanced activation of monocytes incubated with immune-complexes containing anti-CD8α mAb in comparison to immune-complexes with isotype control was also found when TNF release (1376 pg/mL versus 468 pg/mL, p < 0.05 [data not shown]), CD69 upregulation, or CD14 downregulation was measured (Figure 7C).

Blockade of FcR with excess Fc fragments nullified TNF release instigated by isotype and anti-CD8α immune-complexes (Figure 7), suggesting the immune-complex system used is FcγR-dependent, as expected. As CD8α enhancement of TNF production is inhibited by Fc fragments and does not occur with monomeric anti-CD8α mAb, the ability of anti-CD8α mAb to enhance responses of human monocytes appears to depend on co-engagement of FcR.

## Discussion

The present study demonstrates expression of CD8α by monocytic cells, and suggests that CD8α, in addition to co-activating TCR responses may have a previously unacknowledged role in co-activating FcγR responses.

Previous to the present study no evidence existed to demonstrate that human monocytes synthesized CD8α. Human PBMC can appear CD14+/CD8α^hi ^in HIV infected individuals, but this population is due to acquisition of CD14 from monocytes, by T cells, that are mostly CD8α+ [54,55]. A similar effect may explain previous claims of CD8α on monocytes subsequent to dengue virus infection [30]. Other studies have demonstrated that macrophages and dendritic cells can acquire CD8α from T cells, without themselves synthesizing it [28]. Thus, the detection of CD8α protein on monocytes in other studies [29] does not demonstrate that CD8α is expressed by, or more importantly, functional on monocytes.

We provide strong evidence that human monocytes constitutively express CD8α at low levels. Notably, CD8α was observed in monocytes, and the monocytic cell line THP-1 by western blot confirming the presence of CD8α in these cells without possible contribution of FcγR. Moreover, detection of CD8α mRNA in THP-1, CD8α protein on THP-1 and 32 kDa CD8α in lysate of continuously cultured THP-1, demonstrates that these monocytic cells must transcribe CD8α mRNA and translate CD8α protein.

We find that monocytes have less 34 kDa sialylated forms of CD8α compared to T cells. The Mr difference between 32 and 34 kDa forms of CD8α may be due to glycosylation other than sialylation [50] (Figure 5), or palmitoylation of CD8α)[56] at three eligible membrane-proximal cysteines [57]. Monocytes and T cells may express different glycosylation or palmitoylation enzymes that account for predominant accumulation of 32 or 34 kDa CD8α. Phosphorylation of CD8α, or oxidation of the free cysteine in the CD8α Ig-domain may explain pI differences of CD8α remaining after neuraminidase treatment. Sialylation of CD8α [58], like CD8β [42], changes during T cell development, and potentially upon T cell activation. Differential sialylation and palmitoylation of CD8 are known to modulate its ability to bind MHC class I and induce T cell activation [42,43,59] Unfortunately, we cannot relate the differences in CD8α we observe between monocytes and T cells to their comparative ability to bind MHC class I and co-activate signaling.

The ability of CD8 to enhance T cell responses in a TCR-dependent manner was defined over twenty years ago. Since then, CD8α expression by several cell types that lack the TCR such as human NK cells [9], dendritic cells [60], rat macrophages [12] and mast cells [61] has been established. Some evidence has suggested a role for CD8α in apoptosis and survival (e.g. [39]) however a viable model has not emerged to define when and how CD8α activation by its ubiquitous MHC class I ligand may be controlled on these cells that lack the TCR.

MHC class I-like protein TL, which exhibits a restricted expression pattern and a high binding affinity for mouse CD8αα, but not CD8αβ [62] could hypothetically control activation of CD8αα+ monocytes or dendritic cells. One model has proposed that mouse CD8αα may downregulate T cell responses and promote generation of memory T cells by binding TL [63,64] however, evidence that CD8αα has a role in generation of memory T cells is controversial [65-67]. Similarly, while one study suggests an inhibitory role of CD8αα on proliferation and cytotoxicity of T cells (but an enhancement of cytokine release) [68], several studies demonstrate the ability of CD8αα to enhance T cell cytotoxicity and other responses [69-73]. In the absence of a equivalent of TL in humans that binds CD8αα with high affinity, the applicability of this model to humans is even more problematic.

Interestingly, some evidence previous to this report suggested CD8 may be able to co-activate responses of FcγR. Many of the components of FcR and TCR signaling are homologous or interchangeable, such as Fcγ and CD3ζ chain, ZAP-70 and Syk [74,75]., or LAT [76]. For instance, Fcγ and CD3ζ are conserved ancestral duplicates [77] that can substitute for each other in activation of mast cells, γδ or αβ T cells [18,20,23] LAT binds CD8α, and is phosphorylated upon macrophage activation through FcγR [76] or upon Fcγ-dependent activation in mast cells and platelets [78]. A more direct suggestion that CD8 can co-activate Fcγ-chain dependent signaling through TCR was provided by the demonstration of CD8 involvement in TCR-mediated cytotoxicity in Fcγ+, CD3ζη-/- CTL [19]. What is more, Fcγ and Syk are naturally expressed by many mature and immature T cells in humans (less so in mice), and participate in TCR-signaling [23], suggesting that *in vivo *even on T cells CD8 may have a role in activating Fcγ responses[24]. In sum, reasonable although little acknowledged evidence existed that CD8 participated in Fcγ chain-linked responses in T cells. However, no evidence had shown whether CD8 could enhance Fcγ chain responses that were not mediated through TCR thereby expanding this model to include possible functions of CD8 on monocytes, NK cells, dendritic cells, or mast cells.

We and others have previously demonstrated that CD8α on rat Mφ and NK cells signals through Syk and src tyrosine kinases [79], consistent with TCR and FcγR signaling, but in the absence of TCR [80,81] Here, we find that anti-CD8α mAb enhances TNF production of monocytes exposed to immune complexes in an Fc-dependent manner, mirroring the ability of CD8α to enhance T cell activation in a TCR-dependent manner. This evidence suggests CD8α enhances FcγR responses, through at least one of several potential mechanisms. In experiments presented here CD8α may promote initial contact and binding stability of anti-CD8α mAb containing immune-complexes with FcγR. In the same way, CD8α may promote binding of FcγR to MHC class I-expressing cells coated with immune-complexes in cancer, viral infection or autoimmune disease. Alternatively, or in addition, signaling through CD8α may enhance activation of FcγR signaling in our experiments. While we have not directly demonstrated that CD8α signaling enhances FcγR signaling, previous evidence supports this possibility and suggests it merits further investigation. If CD8α signaling can enhance FcγR signaling then hypothetically CD8α may enhance responses of other receptors that both CD3ζ and Fcγ can function with such as NKp30, and NKp46 [14] in NK cells, and FcεRI in mast cells [21].

Our evidence suggests monocyte responses instigated through immune-complexes and FcR can be amplified by co-engagement of CD8α. Interestingly, in rats CD8α+ monocytes and Mφ are found at sites of tissue damage in immune-complex mediated glomerulonephritis [82], arthritis [83], tumor [84], experimental allergic encephalomyelitis (a model of multiple sclerosis) [85], and ischaemia-reperfusion injury [86]. TNF is an important mediator in many of these diseases [87,88] As monocyte TNF production is enhanced by co-activation of CD8α and immune-complexes, CD8α on monocytes may aggravate some autoimmune and acute inflammatory conditions characterized by tissue deposition of immune-complexes.

In summary, we find that human monocytes express CD8α and that monocyte CD8α is differentiable from that on T cells by 2-D electrophoresis. We provide evidence that CD8α on monocytes amplifies responses initiated through FcR, suggesting for the first time a co-activator role for CD8 on cells without the TCR.

## Methods

### Antibodies

Isotype control antibodies were mouse IgG_1 _and IgG_2a _(Sigma, St. Louis, MO), IgG_2a_-FITC, and -PE, (Caltag, Burlingame, CA). Anti-CD8α mAb used were: D9 and 32-M4 (Santa Cruz, Santa Cruz, CA) LT8 (Serotec, Raleigh, NC), B9.11 (Beckman-Coulter Canada Inc., Mississauga, ON), and Nu-Ts/c (Nicherei Corp., Tokyo, Japan). Polyclonal anti-human CD8α Ab (H160) was obtained from Santa Cruz. Anti-CD8α mAb 51.1 (gift of Dr. D. Burshtyn, University of Alberta) and OKT8, and anti-rat MHC class I mAb OX18 (European Collection of Cell Cultures, Salisbury, UK) were purified from hybridoma supernatant by protein G affinity chromatography. Anti-CD8β-antibodies were obtained from Beckman-Immunotech (clone 2ST8.5H7-PE, Mississauga, Canada) and Serotec (clone 5F2). Anti-CD3-FITC and anti-CD14-FITC/PE were obtained from Caltag. Anti-mouse Ig-FITC (STAR70) was obtained from Serotec. Anti-CD69 mAb and matching isotype control were obtained from BD Biosciences. Anti-mouse Ig-HRP was purchased from Pierce (Rockford, IL).

### Cell recovery and culture

The promonocytic cell line THP-1 was maintained in American Type Culture Collection recommended media (RPMI 1640 medium, 2 mM L-glutamine, 1.5 g/L sodium bicarbonate, 4.5 g/L glucose, 10 mM HEPES, 1.0 mM sodium pyruvate 0.05 mM 2-mercaptoethanol, and 10% fetal bovine serum [FBS]). CTL clones [68] were a gift of Dr. Chris Bleackley (University of Alberta).

Human blood (100 mL) was collected into heparanized tubes. Red blood cells were sedimented by addition of 7 mL 6% dextran (Sigma) in RPMI 1640 per 35 mL blood (0.5 h, room temperature). PBMC were enriched on Ficoll-Paque Plus (Amersham Biosciences, Oakville, ON, Canada) and washed three times in PBS. Monocytes were further enriched by three methods. Greater than 80% enriched monocytes were obtained from a Percoll gradient [89] for studies of monocyte activation. Monocytes enriched by Percoll were further purified (>99%) by anti-CD14-PE flow sorting for western blot and RT-PCR analysis. Mφ were differentiated from adherent monocytes (1 h, 37 C) with 500 ng/mL GM-CSF (Biosource, Camarillo, CA) for 3 d.

### Flow cytometry

Cells on ice were blocked with 5% milk, 0.1% bovine serum albumin (BSA) in PBS. In some experiments human Ig (50 μg/mL, Bethyl Laboratories Inc., Montgomery, TX) was used to minimize binding of mAb to FcR. Cells were treated with 10 μg/mL isotype mAb or anti-CD8α mAb, washed three times and incubated with anti-mouse Ig-FITC Ab (1/100, STAR70, Serotec). Anti-CD8β mAb 2ST8.5H7 directly conjugated to PE was used at 10 μg/mL and compared to IgG2a-PE. Cells were washed three times and incubated with 1/10 normal mouse serum before addition of anti-CD14-FITC (1/50).

To analyse the contribution of high affinity FcγRI to anti-CD8α mAb binding to monocytes, PBMC were pre-incubated for 30 min with anti-CD64 mAb clone 10.1 (10 μg/mL, BioLegend, San Diego, CA), which blocks binding of Ig to CD64 [38]. Cells were washed and incubated with IgG2a-PE (10 μg/mL, BD Biosciences) or 32-M4-PE (10 μg/mL, Santa Cruz). Cells were washed and data was collected on a FACScan.

All flow cytometry analysis was performed with WinMDI and CellQuest Pro (BD Biosciences) programs. Monocytes were gated by characteristic FSC/SSC scatter and high expression of CD14.

### Confocal Microscopy

PBMC were adhered to poly-L-lysine coated coverslips for 0.5 h, fixed with 4% paraformaldehyde (10 min) and permeabilized with 0.1% triton-X-100 in PBS (10 min). Cells were blocked (10% FBS, 3% BSA, 30 min) before staining with anti-CD8α mAb (10 μg/mL). Cells were washed three times (5 min, 2 mL PBS) between each reagent. Cells were sequentially incubated with anti-mouse-Ig-rhodamine red (Molecular Probes, Eugene, OR), 1/10 normal mouse serum and anti-CD14-FITC or anti-CD3-FITC (Caltag). Images were obtained using an Olympus FV1000 confocal microscope (Carsen Group, Markham, ON) with Fluoview software.

### Western Blot

Cells were lysed in 1% Triton X-100 for 20 min. Lysate was centrifuged for 5 min at 1000 g to eliminate non-solubilized material. Before loading on gels remaining lysate was diluted 2-fold with Laemmli buffer (BioRad), 2% 2-mercaptoethanol was added and samples were boiled for 5 min. Similar amounts of cell lysate (1–1.5 × 10^6 ^cell equivalents) were loaded per lane on 4–20% SDS-PAGE denaturing gels (Bio-Rad Readygel). Wet protein transfers to PVDF were performed at 100 V for 1 h. PVDF was blocked for 1 h with 5% milk in TBS, 0.1% Tween-20 and subsequently blotted with 0.2 μg/mL primary antibody.

### RT-PCR

Total RNA was isolated using the Qiagen RNeasy kit. RNA was reverse-transcribed with Superscript II reverse transcriptase (Invitrogen, Carlsbad, CA) using oligo(dT) as primers. The cDNA concentration was estimated by absorbance for each sample and diluted to 100 ng/25 ul of reaction volume. The number of cycles was optimized to be in the exponential phase of the reaction by performing the reaction at different cycles. Densitometric analysis of the gels was performed to select optimal PCR cycle numbers. Thereafter, PCR was performed in 20 μl reactions with primer pairs (25 μM) below. Intron-spanning primers of the sequence 5'-TTTCGGCGAGATACGTCTAACCCTGTGC-3' and 5'-TTTAGCCTCCCCCTTTGTAAAACGGGCG-3' were used to generate a CD8α cDNA fragment of 379 bp [70]. Intron-spanning primers generating a 209 bp product for CD8β were 5'-GGTGAAGAGGTGGAACAGGA-3' and 5'-CTTGAGGGTGGACTTCTTGG-3'. A β-actin cDNA fragment of 326 bp was produced using intron-spanning primers of sequence 5'-GGC ATC CTC ACC CTG AAG TA-3' and 5'-AGG GCA TAC CCC TCG TAG AT-3'. PCR amplification was performed for 35 cycles of 1 min at 94 C, 1 min at 60 C and 2 min at 72 C, and a final cycle of 72 C for 10 min to complete polymerization. PCR products were run on a 1.5% agarose gel containing ethidium bromide. Intron-spanning primers were used and samples were treated with 84 U/μL DNase I before RT-PCR to avoid interference of contaminating DNA in purified RNA.

Samples used to amplify CD8α mRNA were also amplified with intron-spanning CD3ζ RT-PCR primers (5'-GCACAGTTGCCGATTACAGA-3' and 5'-GCCACGTCTCTTGTCCAAA-3', 293 base pair product) for 50 cycles, performed as above.

### 2-D electrophoresis

Lymphocytes and monocytes were enriched by collecting non-adherent and adherent cells respectively after 1 h in culture flasks. Lymphocyte and monocyte lysates were prepared using the 2-D cleanup kit (Bio-Rad) and resuspended in IPG strip rehydration buffer (Bio-Rad) with 2% carrier isoelectric point (pI) 3–10 ampholytes (Bio-Rad). Lysates were focused on 7 cm pI 3–10 strips (Bio-Rad).

### Monoclonal antibody affinity chromatography

OKT8 at 5–10 mg/mL in 0.1 M HEPES pH 7.5 was coupled to pre-washed N-hydroxysuccinimidyl-activated agarose beads (Sigma) at 4 C for 1 h. Remaining active sites were blocked by incubating in the presence of 0.1 mL 1 M ethanolamine pH 8 at 4 C for 1 h. Rat cultured mast cell line (RCMC, MHC class I purification) or human thymus (CD8 purification) was lysed with 1% triton X-100 in PBS with Complete Mini anti-protease cocktail tablets (Roche Applied Science, Laval, PQ, Canada). Supernatant remaining after 1000 g, 12,000 g, and 100,000 g centrifugations was loaded on columns. Columns were sequentially washed with 30 volumes lysis buffer, 20 volumes 10 mM Tris 0.5% triton X-100 300 mM NaCl pH 8, 20 volumes 10 mM sodium phosphate 0.5% triton X-100 450 mM NaCl pH 10, and eluted with 0.05 M diethylamine 0.5% triton X-100 650 mM NaCl pH 11.5. 1.5 mL fractions were collected into 50 μL 1 M Tris HCl pH 6.7.

### MALDI-QTOF

Bands were excised and an automated in-gel tryptic digestion was performed on a Mass Prep Station (Water, USA). The gel pieces were de-stained, reduced (DTT), alkylated (Iodoacetamide), digested with trypsin (Promega Sequencing Grade Modified) and the resulting peptides extracted from the gel and analyzed via LC/MS/MS. LC/MS/MS was performed on a CapLC HPLC (Waters, USA) coupled with a Q-ToF-2 mass spectrometer (Waters, USA). Tryptic peptides were separated using a linear water/acetonitrile gradient (0.2% Formic acid) on a Picofrit reversed-phase capillary column, (5 micron BioBasic C18, 300 Angstrom pore size, 75 micron ID × 10 cm, 15 micron tip) (New Objectives, MA, USA), with an in-line PepMap column (C18, 300 micron ID × 5 mm), (LC Packings, CA, USA) used as a loading/desalting column. Protein identification from the generated MS/MS data was done searching the NCBI non-redundant database using Mascot Daemon (Matrix Science, UK). Search parameters included carbamidomethylation of cysteine, possible oxidation of methionine and one missed cleavage per peptide.

### MHC class I binding

PE labeled HLA-A*0201 tetramers assembled [71] with two peptides selected by the SYFPEITHI search engine, *Mycobacterium tuberculosis *antigen 85-B 143–152 (KLVANNTRL) and a diabetes-specific epitope of glutamic acid decarboxylase 114–123 (VMNILLQYVV) were a gift of Dr. John Elliott (University of Alberta). All washes and incubations of cells were done in ice cold PBS with 0.02% NaN_3_. 1 × 10^6 ^human PBMC were incubated 15 min with 1.5 μg tetramers before the addition of CD14-FITC for 15 min. Before flow cytometry analysis cells were washed three times. In some experiments cells were incubated with 40 μg/mL anti-CD8α mAb or isotype control for 30 min prior to addition of tetramers.

### Immune-Complex Stimulation of Monocytes and TNF Measurements

Monocytes were treated with isotype or anti-CD8α mAb (10 μg/mL), or immune-complexes. All mAb were negative for endotoxin using the LAL assay (Sigma-Aldrich). Immune-complexes were prepared by combining isotype mAb or anti-CD8α mAb (10 μg/mL) with anti-mouse Ig (20 μg/mL) for 15 min before addition to monocytes. Monocytes enriched on a Percoll gradient were incubated with immune-complexes for 5 h at 0.2 × 10^6 ^cells/well of a 96 well plate (Becton Dickinson, 35172). Anti-human TNF-PE mAb, fixation and permeabilization buffers, and monensin were from ebioscience (San Diego, CA). Intracellular TNF was detected according to supplier recommendations. Monensin (2 μM) was added 2 h after immune-complexes.

Stimulation of monocytes with immune-complexes was inhibited by pretreating monocytes with 50 μg/mL purified mouse IgG Fc fragment (Jackson Immunoresearch, West Grove, USA) to block binding of immune-complexes to FcR. In these experiments free binding sites of anti-mouse Ig antibody in immune-complexes that might otherwise bind Fc fragments on pretreated monocytes, were pre-blocked with 50 μg/mL purified Fc fragment.

TNF release was measured by ELISA after activation of monocytes for 18 h using 2 μg/mL anti-human TNF mAb as a capture antibody (clone 28401, R&D Systems, Minneapolis, MN) and biotinylated anti-human TNF (BAF210, R&D systems) as a detection antibody. Signal was detected with streptavidin-HRP (Vector Labs, Burlingame, CA) and *o*-phenylenediamine.

### Measurement of CD14 and CD69 Expression

After 18 h activation cells were stained with CD14-FITC/CD69-PE or isotype controls and fixed (ebioscience fixation buffer) before analysis on a FACScan.

## Abbreviations

AM (alveolar macrophage), Mφ (macrophage), geometric mean (Gm), immunoglobulin-like-transcript (ILT), isoelectric point (pI), Linker for Activation of T cells (LAT), peripheral blood mononuclear cell (PBMC)

## Authors' contributions

DG designed and analyzed experiments, wrote the manuscript and performed flow cytometry, western blots, 2-D electrophoresis, confocal microscopy, MHC binding and monocyte activation assays. MMP helped perform confocal microscopy and designed, performed and analyzed RT-PCR results for CD8β and CD3ζ. YS performed 2D blots with polyclonal antibodies and analyzed results. MCYN performed RT-PCR for CD8α and some flow cytometry and western blot. ADB designed and analyzed experiments, revised and approved this document. All authors read and approved the final manuscript.

## Supplementary Material

Additional file 1 Protein recognized by anti-CD8α mAb D9 is CD8α. CD8α was enriched from human thymus lysate by immunoaffinity chromatography with anti-CD8α mAb OKT8. OKT8-reactive fractions were analyzed by western blot with anti-CD8α mAb D9 (left), and silver stain (right) after 2-D electrophoresis. Alignment of western blot and silver stain gels allowed extraction of D9-reactive spots from silver stained gels for peptide sequencing by MALDI-QTOF (lower panel). Click here for file
