# Supplementary material for: CD8α is expressed by human monocytes and enhances FcγR-dependent responses
Source: BMC Immunol. 2007 Aug 1;8:12. doi: 10.1186/1471-2172-8-12 (PMC2000912; doi:10.1186/1471-2172-8-12)
Supplement: Additional file 1 — Protein recognized by anti-CD8α mAb D9 is CD8α. CD8α was enriched from human thymus lysate by immunoaffinity chromatography with anti-CD8α mAb OKT8. OKT8-reactive fractions were analyzed by western blot with anti-CD8α mAb D9 (left), and silver stain (right) after 2-D electrophoresis. Alignment of western blot and silver stain gels allowed extraction of D9-reactive spots from silver stained gels for peptide sequencing by MALDI-QTOF (lower panel). [file 1471-2172-8-12-S1.ppt]

## Slide 1
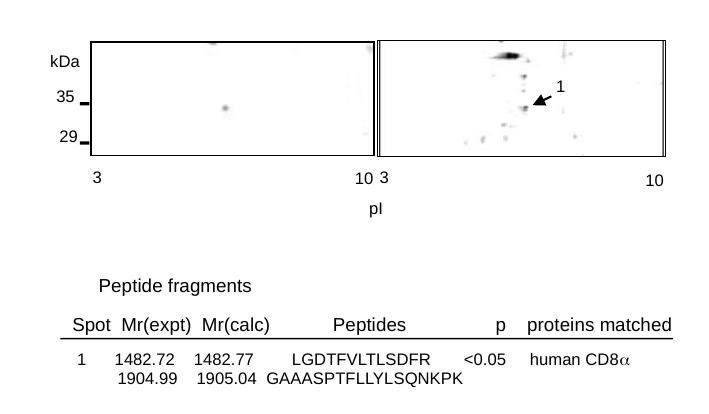

kDa
1
35
29
3
3
10
 10
pI
Peptide fragments
Spot Mr(expt) Mr(calc) Peptides p proteins matched
 1 1482.72 1482.77 LGDTFVLTLSDFR <0.05 human CD8α
 1904.99 1905.04 GAAASPTFLLYLSQNKPK
